# Association between breast cancer risk and disease aggressiveness: Characterizing underlying gene expression patterns

**DOI:** 10.1002/ijc.33270

**Published:** 2020-09-05

**Authors:** Emilio Ugalde‐Morales, Felix Grassmann, Keith Humphreys, Jingmei Li, Mikael Eriksson, Nicholas P. Tobin, Åke Borg, Johan Vallon‐Christersson, Per Hall, Kamila Czene

**Affiliations:** ^1^ Department of Medical Epidemiology and Biostatistics Karolinska Institutet Stockholm Sweden; ^2^ Institute of Medical Sciences University of Aberdeen Aberdeen UK; ^3^ Swedish eScience Research Centre (SeRC) Karolinska Institutet Stockholm Sweden; ^4^ Department of Human Genetics Genome Institute of Singapore Singapore Singapore; ^5^ Department of Surgery, Yong Loo Lin School of Medicine National University of Singapore Singapore Singapore; ^6^ Department of Oncology‐Pathology Karolinska Institutet Stockholm Sweden; ^7^ Department of Clinical Sciences, Division of Oncology and Pathology Lund University Lund Sweden; ^8^ Department of Oncology Lund University Cancer Center Lund Sweden; ^9^ CREATE Health Strategic Centre for Translational Cancer Research Lund University Lund Sweden; ^10^ Department of Clinical Sciences, SCIBLU Genomics Lund University Lund Sweden; ^11^ Department of Oncology Södersjukhuset Stockholm Sweden

**Keywords:** breast cancer, gene expression, prognosis, subtypes, Tyrer‐Cuzick risk score

## Abstract

The association between breast cancer risk defined by the Tyrer‐Cuzick score (TC) and disease prognosis is not well established. Here, we investigated the relationship between 5‐year TC and disease aggressiveness and then characterized underlying molecular processes. In a case‐only study (n = 2474), we studied the association of TC with molecular subtypes and tumor characteristics. In a subset of patients (n = 672), we correlated gene expression to TC and computed a low‐risk TC gene expression (TC‐Gx) profile, that is, a profile expected to be negatively associated with risk, which we used to test for association with disease aggressiveness. We performed enrichment analysis to pinpoint molecular processes likely to be altered in low‐risk tumors. A higher TC was found to be inversely associated with more aggressive surrogate molecular subtypes and tumor characteristics (*P* < .05) including Ki‐67 proliferation status (*P* < 5 × 10^−07^). Our low‐risk TC‐Gx, based on the weighted sum of 37 expression values of genes strongly correlated with TC, was associated with basal‐like (*P* < 5 × 10^−13^), HER2‐enriched subtype (*P* < 5 × 10^−07^) and worse 10‐year breast cancer‐specific survival (log‐rank *P* < 5 × 10^−04^). Associations between low‐risk TC‐Gx and more aggressive molecular subtypes were replicated in an independent cohort from The Cancer Genome Atlas database (n = 975). Gene expression that correlated with low TC was enriched in proliferation and oncogenic signaling pathways (FDR < 0.05). Moreover, higher proliferation was a key factor explaining the association with worse survival. Women who developed breast cancer despite having a low risk were diagnosed with more aggressive tumors and had a worse prognosis, most likely driven by increased proliferation. Our findings imply the need to establish risk factors associated with more aggressive breast cancer subtypes.

## Abstract 2

**What's new?**

The Tyrer‐Cuzick score for assessing breast cancer risk integrates information on lifestyle, reproductive, and familial factors, including *BRCA* mutation status. The relationship between Tyrer‐Cuzick score and specific breast cancer subtypes, however, remains unclear. In this investigation, five‐year, low‐risk Tyrer‐Cuzick gene expression profile, expected to be negatively correlated with risk, was associated with certain, more aggressive breast cancer subtypes, including basal‐like and HER2‐enriched subtypes. Analyses further indicate that genes and biological pathways involved in increased proliferation underlie this association. The findings draw attention to factors relevant to aggressive breast cancer subtypes that are not yet captured in risk assessment tools.

**Abbreviations**
AIMS: Absolute Assignment of Breast Cancer Intrinsic Molecular Subtype
ClinSeq: Clinical Sequencing of Cancer in Sweden
DGE: differential gene expression
ER: estrogen receptor
FDR: false discovery rate
GSEA: gene set enrichment analysis
HER2: human epidermal growth factor receptor 2
IBIS: International Breast Cancer Intervention Study
KARMA: KARolinska MAmmography Project for Risk Prediction of Breast Cancer
Ki‐67: marker of proliferation Ki‐67
LALBA: lactalbumin alpha
LIBRO‐1: Linné‐Bröst 1
low‐risk TC‐Gx: low‐risk Tyrer‐Cuzick gene expression
PGC: progastricsin
PH: proportional hazards
SCAN‐B: The Sweden Cancerome Analysis Network—Breast
TC: Tyrer‐Cuzick score
TCGA: The Cancer Genome Atlas
TNBC: triple‐negative breast cancers

## 1 INTRODUCTION

Breast cancer is a complex disease involving genetic and nongenetic risk factors. Risk assessment tools have been developed to estimate individual breast cancer risk over time.^1^ In particular, the Tyrer‐Cuzick risk score integrates information on established life style, reproductive and familial risk factors.^2^ In order for these tools to help decrease breast cancer mortality through improvement of screening practices, chemoprevention trials or other preventative strategies,^3^ they should be able to predict risk for breast cancer of different subtypes. We have previously observed that women at high risk as predicted by risk assessment tools are more likely to have tumors of more favorable tumor characteristics,^4^ prompting the question whether the association persists for breast cancer subtypes, which are known to differ in their etiology.^5^

Aggressive tumors are characterized by a faster growth rate, greater capability to invade surrounding tissue and metastazise, leading to poorer survival. More aggressive breast cancers tend to be of basal‐like and human epidermal growth factor receptor 2 (HER2)‐enriched intrinsic molecular subtypes,^6^ hormone‐receptor (ER) negative,^7^ higher grade and proliferation status, larger tumor size and lymph node‐positive involvement.^8^, ^9^ Currently, no risk assessment tool is particularly sensitive for predicting risk of aggressive breast cancer subtypes.^10^ The lack of such an algorithm can be partially attributed to a bias because overrepresentation of ER positive and thus less aggressive cancers in most populations where etiology has been studied and risk prediction tools have been established. Therefore, more insights into the biology of breast cancer risk are needed in order to develop preventative approaches that target women at increased risk, particularly of more lethal tumors.^11^

The goal of this study is to investigate the association between Tyrer‐Cuzick risk score and breast cancer subtypes, tumor characteristics and prognosis, and to gain biological understanding of underlying molecular processes by leverage of gene expression data in samples from a clinically representative study population.

## 2 METHODS

Study population consisted of women under the age of 80, diagnosed with primary invasive breast cancer recruited in the Linné‐Bröst 1 (LIBRO‐1) study^12^ or KARolinska MAmmography Project for Risk Prediction of Breast Cancer (KARMA)^13^ studies, in the Stockholm and Skåne region of Sweden. LIBRO‐1 study is a case‐only, population‐based cohort consisting of 5715 women diagnosed with breast cancer in Stockholm during 2001 to 2008. KARMA is a prospective cohort study of 70 877 women with or without breast cancer, recruited in 2011 to 2013, from four mammography units situated in Skåne county and Stockholm.

All LIBRO‐1 and KARMA participants with primary invasive breast cancer diagnosed 2005 to 2015 were considered for inclusion (n = 4598). The cutoff at 2005 was chosen since staining for HER2 and Ki67 immunohistochemistry (IHC) markers was not performed before 2005. In total, 2632 cases with complete information on all the IHC markers, needed to derive surrogate molecular subtypes, were eligible for this study.

### 2.1 Tumor characteristics, surrogate molecular subtypes and survival

Information on molecular markers was retrieved from medical and pathology records at treating hospitals. Percent of estrogen receptor (ER) and progesterone receptor (PR) staining was dichotomized into positive or negative status (positive if ≥10%, otherwise negative) during this period. HER2 status was dichotomized according to the Swedish Society of Pathology's guidelines, as being negative if protein expression showed 0 or 1+, or higher with no confirmed gene amplification by FISH, and as being positive if FISH showed gene amplification. Proliferation marker Ki67 was measured in hotspot regions according to contemporary guidelines and reported as percent staining (low if <20% and high otherwise). Surrogate molecular subtypes were derived from ER, PR and HER2 status; Ki67 percentage values; age at diagnosis, using a subtype classifier based on a random forest algorithm trained to predict breast cancer molecular subtypes.^14^

Data on clinical tumor characteristics and prior breast cancer diagnoses were obtained through the Swedish National Cancer Register^15^ and the Stockholm‐Gotland Regional Breast Cancer Quality Register^16^ using the Swedish personal identity numbers.^17^ Lymph node involvement was dichotomized as being positive or negative. Tumor size diameter was measured in millimeters. Tumor grade was recorded using the Nottingham Histologic Grade system.

Date of death was obtained from the Swedish Cause of Death Register (linkage performed on 6 October 2017). Breast cancer‐specific events were identified in cases with cause of death code “C50*.” The quality of the registry is high. A high correlation (95.9%) between hospital discharge diagnosis and underlying cause of death from death certificates for malignant breast neoplasms has been observed.^18^

### 2.2 Tyrer‐Cuzick risk score

Individual 5‐year TC was computed using the International Breast Cancer Intervention Study (IBIS) tool version 7 (http://www.ems-trials.org/riskevaluator/), based on the Tyrer‐Cuzick model.^2^ The model included risk factors of age, age at menarche, age at first child, menopause, length, weight, hormone‐replacement therapy use and previous benign breast disease (eg, hyperplasia, atypical hyperplasia, lobular cancer in situ). The score also includes first‐/second‐degree family history of breast and ovarian cancer, Ashkenazy descent and BRCA mutation status. Information on these variables was available from a self‐reported Web‐based questionnaire during study recruitment, with 95% to 100% completeness. BRCA1/2 mutation status was defined based on the carriership of at least one rare protein‐truncating variant, as previously described.^19^ TC scores were calculated at age of first breast cancer diagnosis. Variables were coded according to the Tyrer‐Cuzick protocol.

### 2.3 Gene expression data sets

Two tumor RNA‐sequencing data sets comprising LIBRO‐1 and KARMA participants with breast cancer were analyzed in a discovery‐validation setting. The discovery data set consisted of 296 participants that were sequenced under the Clinical Sequencing of Cancer in Sweden (ClinSeq) project.^20^ The validation data set consisted of 376 participants sequenced under The Sweden Cancerome Analysis Network—Breast (SCAN‐B) initiative.^21^ Sample preparation, sequencing protocol and gene expression quantification are described in the supplementary methods.

An independent RNA‐seq data set consisted of breast cancer expression data from The Cancer Genome Atlas (TCGA).^22^ RNA‐seq expression data (HTseq counts), together with patient clinical information, was retrieved using the GDC Data Transfer Tool on 7 November 2018. In total, 975 primary invasive breast carcinomas with age at diagnosis between 26 and 90 years old were included in this study.

### 2.4 PAM50 molecular subtypes

PAM50 molecular subtypes were computed on the discovery, validation and independent data sets from RNA‐seq normalized counts using a research‐based subtype predictor, the Absolute Assignment of Breast Cancer Intrinsic Molecular Subtype (AIMS) method^23^ version 1.12.0 in R.

### 2.5 Correlation of gene expression levels with TC

Regression analyses were used to correlate tumor gene expression with TC. TC score was available for 259 (87.5%) of women in the discovery data set and ranged from 0.1% to 9.5% with a mean of 2.0% in the validation data set, TC was obtained for 313 (83.24%) women and ranged from 0.4% to 7.1% with a mean of 2.1%. In order to capture effects by lower risk, the 5‐year TC risk score was transformed by subtracting its value from zero (ie, creating a negative TC), so that gene‐level effect sizes (beta coefficients) represent expression changes related to a 1‐percentage decrease on the TC scale. The regression analyses were performed using the quasi‐likelihood (QL) dispersion estimation and hypothesis testing method implemented in the edgeR package^24^, ^25^ in R. Under this methodology, RNA‐seq count‐based data are modeled using a negative binomial (NB) distribution. Regressions were fitted based on the NB general linear model using the *glmQLFit* (robust = *T*) function, and beta coefficients were obtained using the QL *F*‐test with the *glmQLFTest* function. Genes with a mean counts per million value of <0.5 were considered weakly expressed and therefore were not included in the analysis. Differences in library composition, for example, total number of counts per sample, were normalized using the trimmed mean of *M*‐valued method.^26^

### 2.6 Low‐risk TC‐gene expression profile

A low‐risk Tyrer‐Cuzick gene expression (TC‐Gx) profile was calculated for each individual in the discovery, validation and TCGA expression data sets, as the weighted sum of gene expression values (weighted by the beta coefficients, which are on the scale of a per 1% decrease in TC). The profile was based on genes found to be significantly correlated with the TC score through regression analysis in the discovery data set. We controlled the false discovery rate (FDR) to be lower than 0.05, and significantly associated genes were required to have an absolute effect size larger than 1.5‐fold (ie, beta coefficient larger than ±log_2_[1.5]). Effect size beta estimates, corresponding to a 1% decrease in 5‐year TC risk, were used to weight the normalized and log_2_‐transformed expression values. The low‐risk TC‐Gx therefore represents a weighted sum of gene expression values, which is expected to be negatively correlated with breast cancer risk. Genes with low expression values (ie, below 0.5 mean counts per million) in the validation data set were not included in the final TC‐Gx.

### 2.7 Statistical methods

Statistical analysis was performed in R (version 3.5.2). All statistical tests were two‐sided, with an alpha level set at 0.05, or as specified otherwise. We summarized association between continuous exposures (eg, TC score and low‐risk TC‐Gx) and outcome variables, one at a time. Binary outcomes such as hormonal status were analyzed using unconditional binomial logistic regression, and categorical outcomes such as molecular subtypes and tumor grade were modeled with unconditional multinomial logistic regressions using the R “nnet” package. The TC score was treated as a continuous linear score, and odds ratios are reported in terms of per one‐percentage increase. The TC‐Gx was treated as a standardized continuous linear score, and odds ratios are reported in terms of per 1 SD increase.

#### 2.7.1 Gene Set Enrichment Analysis

To test for gene sets enriched for overall gene expression correlated with TC, we performed gene set enrichment analysis (GSEA) methods that do not rely on predefined significance thresholds (ie, no *P* value cutoff is applied), using the workflow implemented in the Piano^27^ R package. Gene sets were defined using the Molecular Signature Database (MSigDB) hallmark collection, consisting of 50 hallmark gene sets curated from a number of “founder” gene sets.^28^ A gene set was considered enriched if affected by the constituent genes compared with the rest of the genes. Detailed input and workflow settings are described in supplementary methods.

#### 2.7.2 Survival analysis

Multivariate Cox proportional hazard regression models were used to estimate 10‐year breast cancer‐specific survival using the “survival” R package, with time since diagnosis as the underlying time scale. For this analysis, we combined patients from the validation (ClinSeq) and discovery (SCAN‐B) samples with complete information on survival (n = 661). Of these, 416 (62.8%), were prevalent cases. Kaplan‐Meier survival curves were visualized using the “ggkm” R package. Time at risk was considered from date of study entry (eg, blood draw and left truncation) until date of breast cancer death, or censoring, due to any cause of death or end of follow‐up (truncated at 10 years), whichever occurred first. For survival analysis, the low‐risk TC‐Gx was dichotomized according to the mean of the distribution (ie, above vs below the mean distribution). Cox proportional hazards (PH) models were adjusted for data set, year and age at diagnosis. Additional Cox PH models were further adjusted for *MKI67* and *ESR1* log_2_‐gene expression levels and PAM50 subtypes.

## 3 RESULTS

### 3.1 Association between breast cancer risk and disease aggressiveness

In our case‐only cohort of 2474 invasive breast cancer patients for whom information on established breast cancer risk factors had been collected (Supplementary Table 1), the 5‐year breast cancer risk, TC score, ranged from 0.1% to 10.8%, with a mean of 2.0%. We found that women with a higher TC (as per 1% increase in TC) were less likely to be diagnosed with basal‐like (*P* = 1.39 × 10^−6^) and HER2‐enriched (*P* = 1.39 × 10^−6^) surrogate molecular subytpes of breast cancer (*P* < .05), ER‐negative (*P* < 1 × 10^−3^), HER2‐positive (*P* < .05), lymph‐node positive (*P* < 1 × 10^−3^), higher tumor grade (*P*‐trend < 1 × 10^−06^) and higher Ki‐67 proliferation status (*P* < 5 × 10^−7^) (Table 1). Exclusion of women with family history of breast cancer, and BRCA mutation status, did not affect the observed inverse association between TC and disease aggressiveness (data not shown).

**TABLE 1:** Association of 5‐year breast cancer risk (TC score) with surrogate molecular subtypes and tumor characteristics in 2474 LIBRO‐1/KARMA cases

Outcome	n	%	OR	95% CI	*P* value
**Surrogate subtype**					
Luminal A	1802	72.84	Ref		
Basal‐like	153	6.18	**0.599**	**0.487**, **0.738**	**1.39E−06**
HER2‐enriched	272	10.99	**0.867**	**0.771**, **0.975**	**1.72E−02**
Luminal B	247	9.98	0.918	0.818, 1.030	1.44E−01
**ER status**					
Positive	2116	85.6	Ref		
Negative	356	14.4	**0.825**	**0.736**, **0.918**	**6.11E−04**
**PR status**					
Positive	1697	68.65	Ref		
Negative	775	31.35	0.961	0.894, 1.030	2.66E−01
**HER2 status**					
Negative	2174	88.34	Ref		
Positive	287	11.66	**0.872**	**0.774**, **0.975**	**2.04E−02**
**Lymph node status**					
Negative	2135	87.79	Ref		
Positive	297	12.21	**0.809**	**0.713, 0.910**	**6.65E−04**
**Grade**					
Well differentiated	448	18.9	Ref		
Moderately differentiated	1210	51.05	0.959	0.884, 1.039	3.07E−01
Poorly differentiated	712	30.04	**0.777**	**0.702, 0.861**	**1.25E−06**
				*P*‐trend=	**5.03E−07**
**Tumor size**					
<20 mm	1521	62.44	Ref		
≥20 mm	915	37.56	0.950	0.886, 1.016	1.38E−01
**Ki‐67**					
Low (<20%)	1364	55.31	Ref		
High (≥20%)	1102	44.69	**0.831**	**0.774, 0.890**	**2.24E−07**

*Note:* Odds ratios with 95% CI are shown per 1‐percentage point increase in the 5‐year TC. Boldface type indicates associations significant at *α* = .05. Unconditional regression analysis for association of 5‐year TC score with surrogate molecular subtypes, and tumor characteristics in 2474 LIBRO‐1/KARMA cases.

Abbreviations: ER, estrogen receptor; OR, odds ratio; PR, progesterone receptor.

### 3.2 Definition of a low‐risk TC‐Gx profile

**FIGURE 1:**
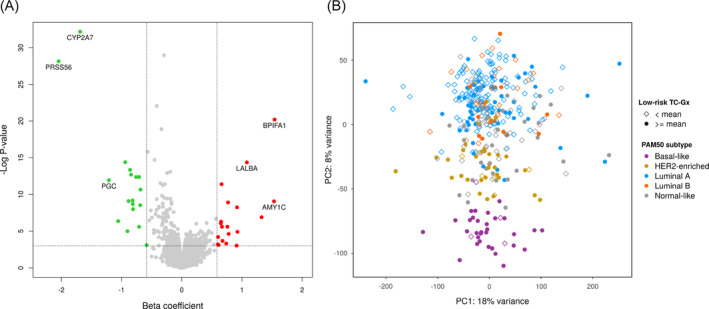
Discovery of genes correlated with low 5‐year risk for breast cancer as estimated by the Tyrer‐Cuzick risk model and correspondence of the low‐risk TC‐Gx with the PAM50 subtypes. A, Volcano plot showing differential expression for low TC in the discovery data set. Genes are displayed by strength of association (beta coefficient, *β*, as per 1% decrease in TC) and statistical significance (−log *P* value). An individual‐level TC‐Gx profile was computed based on 37 top genes (FDR < 0.05 and *β* > ±log_2_[1.5]) marked in green (downregulated) and red (upregulated). Gene names are shown for the genes with the strongest association (*P* value <1 × 10^−8^ and *β* > ±log_2_ (2)). B, Principal component analysis (PCA) plot showing distribution of validation samples based on whole transcriptomic profiles. Samples are labeled by PAM50 subtype and by low‐risk TC‐Gx dichotomized according to the mean of the distribution. Tumors with an increased low‐risk TC‐Gx profile (eg, ≥ mean distribution) were more common among basal‐like and HER2‐enriched subtypes, and less likely labeled as luminal and normal‐like subtypes

Using our discovery data set, we identified 37 top genes significantly correlated with the TC score (FDR < 0.05 and *β* > 1.5‐fold) (Figure 1A). Based on these genes (Supplementary Table 2), for each individual we computed a low‐risk TC‐Gx profile, as a weighted sum of normalized gene expression values, defined in such a way that the profile is negatively correlated with breast cancer risk (Methods and Supplementary Figure 1). Tumors with an enriched low‐risk TC‐Gx (above the mean distribution) tended to overlap with basal‐like and HER2‐enriched subtypes (Figure 1B).

### 3.3 Association between low‐risk TC‐Gx profile and PAM50 subtypes

The low‐risk TC‐Gx was associated with more aggressive PAM50 subtypes in our validation and discovery data sets (Table 2). In particular, our low‐risk TC‐Gx was consistently associated with a higher probability for basal‐like (*P* < 5 × 10^−13^) and HER2‐enriched tumors (*P* < 5 × 10^−7^). Importantly, the low‐risk TC‐Gx computed in an independent data set from TCGA was associated with more aggressive PAM50 subtypes and tumor characteristics (Table 3).

**TABLE 2:** Association of the low‐risk TC‐Gx profile with PAM50 subtypes: discovery and validation data set

	Discovery (*n* = 296)					Validation (n = 376)				
PAM50 subtype	n	%	OR	95% CI	*P* value	n	%	OR	95% CI	*P* value
Luminal A	91	30.74	Ref			180	47.87	Ref		
Basal‐like	29	9.80	**12.111**	**6.237**, **23.515**	**1.74E−13**	36	9.57	**13.206**	**7.099**, **24.57**	**3.72E−16**
HER2‐enriched	47	15.88	**4.217**	**2.443**, **7.279**	**2.36E−07**	40	10.64	**4.791**	**2.947**, **7.791**	**2.67E−10**
Luminal B	81	27.36	1.261	0.757, 2.099	3.73E−01	39	10.37	1.235	0.784, 1.944	3.62E−01
Normal‐like	48	16.22	**1.819**	**1.033**, **3.204**	**3.82E−02**	81	21.54	1.336	0.946, 1.888	1.00E**−**01

*Note:* Odds ratios with 95% CI are shown per 1‐SD increase in the TC‐Gx profile. Boldface type indicates associations significant at *α* = .05. Unconditional multinomial regression analysis for the association of the low‐risk TC‐Gx profile with PAM50 subtypes in the discovery and validation data set.

**TABLE 3:** Association of the low‐risk TC‐Gx profile with PAM50 subtypes and tumors characteristics: independent TCGA data set

Outcome	n	%	OR	95% CI	*P* value
**PAM50 subtype**					
Luminal A	354	36.31	Ref		
Basal‐like	167	17.13	**8.060**	**5.95**, **10.919**	**2.27E−41**
HER2‐enriched	102	10.46	**3.931**	**2.921**, **5.291**	**1.70E−19**
Luminal B	287	29.44	1.074	0.884, 1.305	4.74E**−**01
Normal‐like	65	6.67	**1.495**	**1.068**, **2.092**	**1.90E−02**
**ER status**					
Positive	724	77.52	Ref		
Negative	210	22.48	**4.037**	**3.264**, **5.063**	**1.04E−35**
**PR status**					
Positive	626	67.24	Ref		
Negative	305	32.76	**2.250**	**1.919**, **2.658**	**1.58E−22**
**HER2 status** ^a^					
Negative	313	82.59	Ref		
Positive	66	17.41	1.118	0.857, 1.459	4.10E**−**01
**Lymph node** ^b^					
Negative	404	49.33	Ref		
Positive	415	50.67	0.954	0.831, 1.094	4.97E**−**01
**Stage** ^**c**^					
I	162	17.33	Ref		
II	556	59.47	1.277	1.064, 1.532	**8.47E−03**
III	217	23.21	1.191	0.965, 1.469	1.03E**−**01
				*P*‐trend=	1.73E**−**01

*Note:* Odds ratios with 95% CI are shown per 1‐SD increase in the TC‐Gx. Boldface type indicates associations significant at *α* = .05. Unconditional regression analysis for the association of the low‐risk TC‐Gx profile with PAM50 subtypes and tumor characteristics in an independent data set (n = 975) from TCGA.

Abbreviations: ER, estrogen receptor; OR, odds ratio; PR, progesterone receptor.

^a^ FISH method.

^b^ Dichotomized number of lymph node examined under histological evaluation.

^c^ Stage I: stage I, IA and IB; Stage II: stage II, IIA and IIB; Stage III: stage III, IIIA, IIIB, IIIC.

### 3.4 Gene set enrichment analysis

We found that 15 out of the 50 MSigDB gene sets were significantly enriched for overall gene expression by lower TC, under at least one directionality (Table 4). Proliferation and signaling processes were the most common pathways likely to be affected by upregulated genes (ie, genes associated with lower TC risk). Proliferation gene sets were related to E2F and MYC targets, G2M checkpoint and mitotic spindle. Signaling gene sets included estrogen response, mTORC1 and WNT beta catenin signaling.

**TABLE 4:** Gene set enrichment analysis results of overall differential expression by lower breast cancer risk

						Directionality class				
Gene set name	Category	N	n	n(dn)	n(up)	Dist(dn)	Mix(dn)	Nondir	Mix(up)	Dist(up)
E2F_TARGETS	Proliferation	200	133	24	109	1.00E+00	9.95E**−**01	9.82E**−**01	7.71E**−**01	**1.00E−03**
G2M_CHECKPOINT	Proliferation	200	123	22	101	1.00E+00	9.95E**−**01	9.82E**−**01	7.33E**−**01	**1.00E−03**
MITOTIC_SPINDLE	Proliferation	200	121	27	94	1.00E+00	9.95E**−**01	9.13E**−**01	7.11E**−**01	**1.00E−03**
MYC_TARGETS_V1	Proliferation	200	118	7	111	1.00E+00	9.95E**−**01	1.22E**−**01	1.05E**−**01	**1.00E−03**
MYC_TARGETS_V2	Proliferation	58	40	3	37	1.00E+00	9.95E**−**01	**5.00E−03**	**1.00E−02**	**1.00E−03**
ESTROGEN_RESPONSE_EARLY	Signaling	200	100	28	72	1.00E+00	9.95E**−**01	9.13E**−**01	7.11E**−**01	**2.14E−03**
MTORC1_SIGNALING	Signaling	200	95	25	70	1.00E+00	9.95E**−**01	9.83E**−**01	9.39E**−**01	**6.43E−03**
ESTROGEN_RESPONSE_LATE	Signaling	200	99	32	67	1.00E+00	9.95E**−**01	9.13E**−**01	6.63E**−**01	**6.87E−03**
UNFOLDED_PROTEIN_RESPONSE	Pathway	113	60	19	41	1.00E+00	9.95E**−**01	9.07E**−**01	3.67E**−**01	**8.33E−03**
UV_RESPONSE_UP	DNA damage	158	81	30	51	1.00E+00	9.95E**−**01	9.82E**−**01	6.47E**−**01	**3.30E−02**
WNT_BETA_CATENIN_SIGNALING	Signaling	42	25	8	17	1.00E+00	9.95E**−**01	9.13E**−**01	4.34E**−**01	**4.04E−02**
GLYCOLYSIS	Metabolic	200	91	30	61	1.00E+00	9.95E**−**01	9.83E**−**01	9.17E**−**01	**4.46E−02**
BILE_ACID_METABOLISM	Metabolic	112	54	35	19	**2.00E−02**	9.95E**−**01	9.13E**−**01	9.17E**−**01	1.00E+00
COMPLEMENT	Immune	200	59	39	20	**2.75E−02**	9.95E**−**01	9.83E**−**01	9.94E**−**01	1.00E+00
XENOBIOTIC_METABOLISM	Metabolic	200	90	55	35	**3.67E−02**	9.95E**−**01	9.83E**−**01	9.99E**−**01	1.00E+00

*Note:* Top‐ranked molecular signature (MSigDB) hallmark gene sets significantly enriched for overall gene expression correlated to TC (ie, as per 1% decrease in TC), in at least one directionality class. Upregulated classes consist of enrichment for genes negatively associated with TC, while downregulated classes do so for genes positively associated with TC. The median‐adjusted *P* value from six GSEA methods (Wilcoxon rank‐sum test, tail strength, mean, median, sum, reporter features and Stouffer's method) is shown. Boldface type indicates associations significant at *α* = .05.

Abbreviations: GSEA, gene set enrichment analysis; N, number of gene set constituent genes; n; number of constituent genes included in GSEA tests; dn, downregulated; up, upregulated; Dist, distinct‐directional; Mix, mixed‐directional; Nondir, nondirectional.

### 3.5 Breast cancer‐specific survival

**FIGURE 2:**
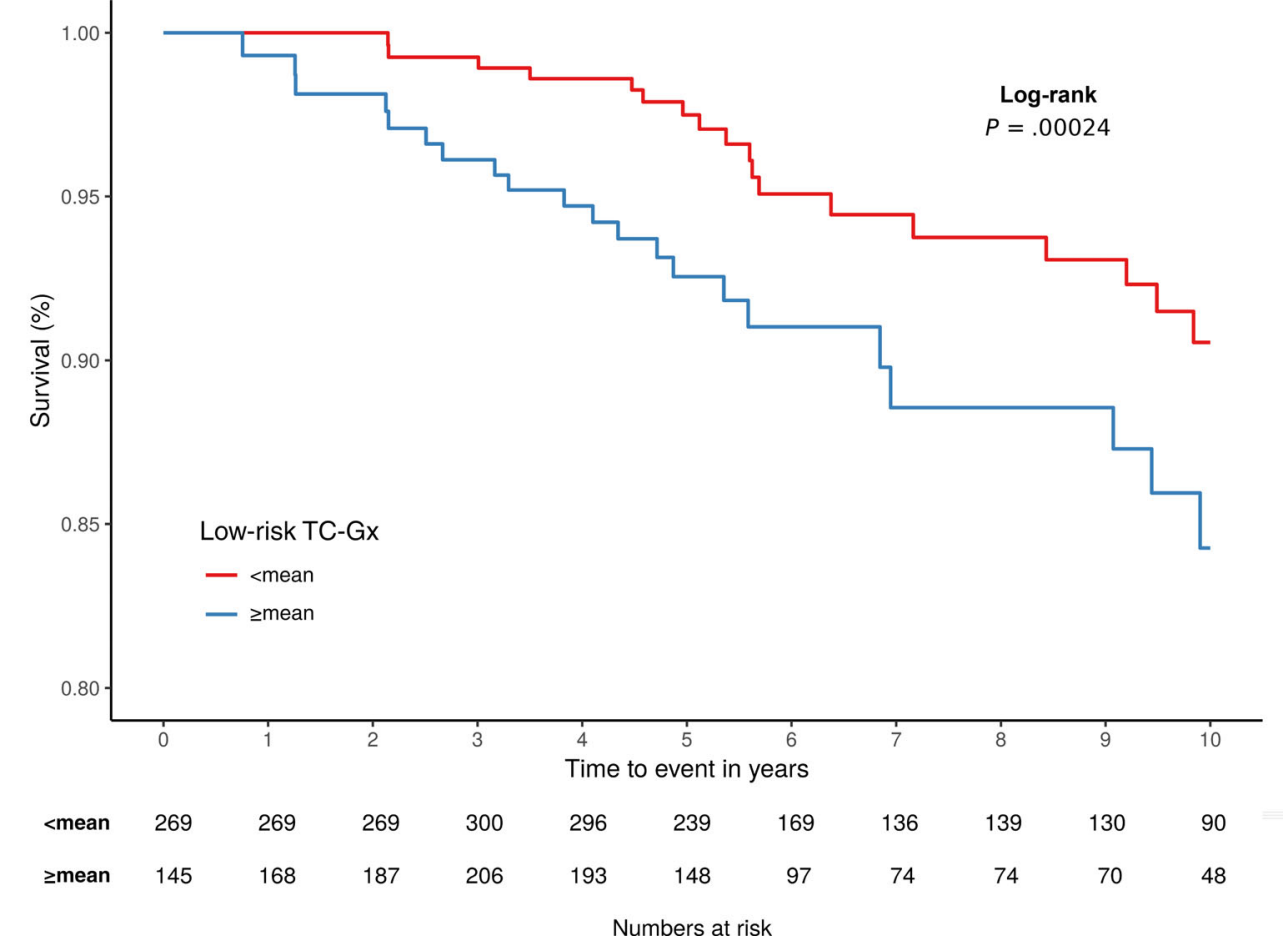
Kaplan–Meier plot showing 10‐year breast cancer‐specific survival by low‐risk TC‐Gx in 661 women from the discovery and validation data set. Log‐rank *P* value obtained from Cox‐model adjusted for data set, age and year of diagnosis, is shown. The low‐risk TC‐Gx was dichotomized according to the mean of the distribution (ie, ≥mean vs <mean distribution)

We observed 39 events from 661 patients in our discovery‐validation data set. Tumors with an increased low‐risk TC‐Gx were found to be associated with worse survival using Cox models adjusted for data set, age and year of diagnosis (log‐rank *P* value = .00024; Figure 2); (HR: 2.29; 95% CI, 1.21‐4.35) (Table 5). Additional adjustment for proliferation status (defined as log_2_ *MKI67* expression levels) attenuated the association, similar to adjustment for PAM50 subtypes, while adjustment for estrogen receptor status (defined as log_2_ *ESR1* expression levels) did not change, substantially, the survival estimates.

**TABLE 5:** HR and corresponding 95% CI for the association of low‐risk TC‐Gx with 10‐year breast cancer‐specific survival: discovery and validation data set combined

Low‐risk TC‐Gx	n	*n*event	HR	95% CI	*P* value
<mean	398	18	Ref		
≥mean	263	21	**2.294**	**1.210**, **4.347**	**.011**
+PAM50			1.751	0.849, 3.610	.129
+MKI67			1.780	0.895, 3.539	.100
+ESR1			**2.234**	**1.077**, **4.636**	**.031**

*Note:* Results from Cox proportional hazards models adjusted for data set (ie, discovery/validation), age and year at diagnosis. Separate models were fitted with additional adjustment for PAM50 subtypes, log_2_(*MIK67*) or log_2_(*ESR1*), respectively. The low‐risk TC‐Gx was dichotomized according to the mean of the distribution. Boldface type indicates associations significant at *α* = .05.

Abbreviations: CI, confidence interval; HR, hazard ratio; TC‐Gx, TC gene expression.

## 4 DISCUSSION

A high breast cancer risk as measured by 5‐year TC score was associated with less aggressive breast cancer. In a subset of patients, a low‐risk TC‐Gx profile was found to be associated with more aggressive PAM50 subtypes (basal‐like and HER2‐enriched) and with worse breast cancer‐specific survival. In addition, differential gene expression associated with low breast cancer risk was found to be related to key biological processes involved in tumor proliferation and oncogenic signaling pathways. This may explain why we observe that some patients, despite having lower risk of breast cancer, tend to develop more aggressive tumors. To our knowledge, this is the first epidemiological study utilizing gene expression data to provide molecular biology insights into the relation between breast cancer risk and disease aggressiveness.

The lack of established risk factors associated with more aggressive subtypes could explain why lower TC scores are more frequent in patients with aggressive tumor characteristics. Several of the lifestyle‐ and reproductive risk factors determining the TC risk score have been shown to be positively associated with ER positive and thus less aggressive breast cancer as previously reviewed,^29^, ^30^ and this is consistent with our findings. Therefore, risk factors linked to the etiology of basal‐like, HER2‐enriched and fast growing tumors would need to be pinpointed and taken into account in order for risk assessment tools to accurately predict risk to develop breast cancer, including the aggressive subtypes.

Our low‐risk TC‐Gx profile included genes known to be biomarkers of specific breast cancer subtypes. In particular, lactalbumin alpha (*LALBA*) and progastricsin (*PGC*) were replicated with strong evidence of association with breast cancer risk. Higher RNA expression of *LALBA* has been found to be associated with more aggressive breast cancer, such as triple‐negative breast cancers (TNBC),^31^ while *PGC* expression has been associated with more favorable tumor characteristics and prognosis related to ER‐positive disease.^32^, ^33^, ^34^ Consistently, we observed LALBA to be associated with lower breast cancer risk and PGC with higher risk. This may explain why our low‐risk TC‐Gx profile was associated with aggressive PAM50 subtypes, despite that none of the genes contributing to the low‐risk TC‐Gx are part of the genes defining the PAM50 subtypes.

Our results suggest that the association between lower risk of breast cancer and more aggressive disease is likely due to altered biological processes involved in proliferation and oncogenic signaling pathways. We found enrichment for proliferation‐related gene sets related to E2F and MYC targets and mitotic spindle processes. E2F transcription factors have been found overexpressed in breast cancer tumors and associated with prognosis in TNBC,^35^ and to be critical in HER2+ tumor development and progression.^36^ MYC overexpression is associated with basal‐like tumors and shorter metastasis‐free survival in Luminal A lymph‐node positive tumors,^37^ is constitutively overexpressed in HER2+ tumors through loss of p53,^38^ and activation of MYC downstream pathways is thought to be related to aggressive tumors with acquired therapy resistance.^39^ With regard to enriched signaling‐related gene sets, these represented involvement in estrogen response, mTORC1 and WNT beta catenin pathways. The former two have been suggested to harbor potential therapeutic targets in TNBC.^40^, ^41^ Interestingly, we found that patients with tumors whose expression pattern more closely resembles low‐risk tumors (as defined by our low‐risk TC‐Gx profile), had a worse breast cancer‐specific survival, which was partially explained by proliferation status and PAM50 subtypes, but not by estrogen‐receptor status.

Some limitations and methodological considerations should be discussed for this study. A considerable proportion of ki67 proliferation data was missing in our validation data set. We addressed this issue by using *MKI67* expression in the survival analysis, which was found to be moderately correlated with ki67 percent staining (*r* = 0.64). Adjustment for other proliferation genes, that is, *AURKA* and *PCNA*, yielded similar results (data not shown). Also, we lacked information on breast cancer‐specific survival in the TCGA data set; therefore, the negative association of our low‐risk TC‐Gx profile with survival time needs to be further replicated.

In conclusion, our results suggest that gene expression patterns associated with low breast cancer risk are related to tumors of more aggressive subtypes, in which deregulation of proliferative and oncogenic signaling pathways can lead to worse prognosis. Importantly, inquiry into molecular and pathological features of breast cancer in relation to known risk factors is an important approach toward better understanding of complex etiology of breast cancer. This is in accordance with the necessity to incorporate subtype‐specific risk factors into current assessment tools in order to identify women at increased risk of aggressive breast cancer and to contribute to effectively decrease disease burden.

## CONFLICT OF INTEREST

The authors declare no conflict of interest.

## ETHICS STATEMENT

Ethical approvals for the LIBRO‐1 and KARMA studies were granted from the regional ethical review board at Karolinska Institutet. All women gave written informed consent to participate in the study, to the retrieval of information from medical records, national registries and mammographic images; donated blood at enrollment for genetic analysis and answered a detailed questionnaire about background and lifestyle risks factors. The study was conducted in accordance with the Declaration of Helsinki.

## Supporting information


**Appendix** **S1:** Supporting information

## Data Availability

Access to data from LIBRO‐1 and KARMA participants cannot be shared due to IRB requirements but can be shared upon reasonable request to the PIs (Kamila Czene and Per Hall). Data access to the KARMA study can be requested from https://karmastudy.org/data-access/.
