# Supplementary material for: Association between breast cancer risk and disease aggressiveness: Characterizing underlying gene expression patterns
Source: Int J Cancer. 2020 Sep 5;148(4):884–94. doi: 10.1002/ijc.33270 (PMC7818270; doi:10.1002/ijc.33270)
Supplement: Supplementary file 1 — Appendix S1: Supporting information [file IJC-148-884-s001.pdf]

SUPPLEMENTAL MATERIAL

Association between breast cancer risk and disease aggressiveness: characterizing underlying gene expression patterns

Emilio Ugalde-Morales, Felix Grassmann, Keith Humphreys, Jingmei Li, Mikael Eriksson, Nicholas P. Tobin, Åke Borg, Johan Vallon-Christersson, Per Hall, Kamila Czene

Table of contents:

Supplementary Materials and Methods
Supplementary Table 1. Descriptive table of risk factors for 2,474 LIBRO-1/KARMA participants.
Supplementary Table 2. List of the 37 genes associated with breast cancer risk (TC score) in the discovery dataset, included in the low-risk TC-Gx.
Supplementary Figure 1. Scatterplot showing correlation between the low-risk TC-Gx and the TC score in the discovery dataset.
Supplementary references

Supplementary Materials and Methods

Gene expression data and quantification

Two tumor RNA-sequencing data sets comprised of LIBRO-1 and KARMA participants diagnosed with primary invasive breast cancer were used in a discovery-validation setting. The discovery dataset included 307 participants that were sequenced under the Clinical Sequencing of Cancer in Sweden (Clinseq) project.(1) The validation dataset consisted of 428 participants sequenced under The Sweden Cancerome Analysis Network – Breast (SCAN-B) initiative.(2) Clinseq experimental protocols were approved by the Regional Ethical Review Board in Stockholm (Reference number: 2013/1833-31/2). The SCAN-B project was approved by the Regional Ethical Review Board in Lund (diary numbers 2007/155, 2009/658, 2009/659, 2014/8), the county governmental biobank center, and the Swedish Data Inspection group (diary number 364-2010).

RNA-seq libraries from the discovery dataset (Clinseq) were generated from 1 micro gram of high quality total RNA (RIN > 8, assessed using bioanalyzer) and rRNA was depleted using RiboZero (Illumina, US). For each sample, stranded RNA-seq libraries were constructed using a TruSeq Stranded Total RNA Library Prep Kit (Illumina, US). Then 2 × 101 paired-end sequencing libraries, at a median of 33 million reads per library, were generated on an Illumina HiSeq 2500 (Illumina, US) at the Science for Life Laboratory (Stockholm, Sweden). From a total of 307, 296 samples from unique individuals were included in this study after quality control and removing potential outliers. For the validation dataset (SCAN-B), high quality RNA samples (RIN/RQS > 7), were used to construct RNA-seq libraries from poly-A enriched RNA using the SCAN-B dUTP library protocol (see full description in Ref.(2)). Paired-end libraries of 50 bp length were sequenced on an Illumina HiSeq2000 instrument. After exclusion, 376 out of 428 samples were available for our validation analysis. These tumor samples had a minimum of more than 5 million successfully aligned paired-reads and less than 60% duplication level estimated for the aligned reads.

Gene expression quantification was performed in two steps: first by computing transcript-level estimates and then by gene-level aggregation. Transcript-level expression estimates were quantified for each sample using Salmon(3) version 0.9.1 (quasi-mapping based mode). Index was built on the reference transcriptome GRCh38 downloaded from ENSEMBL database (ftp://ftp.ensembl.org/pub/release-92/fasta/homo_sapiens/cdna/), using the following parameters: --type quasi -k 19. The *tximportData* package as implemented in R version 3.5.0 was used to import the files containing the transcript level expression values obtained from Salmon. Gene-level aggregation was performed using the *tximport* package(4) v1.8. Gene-to-transcript IDs were extracted from Homo_sapiens.GRCh38.92.gtf annotation file using *rtracklayer* and

tidyverse packages to be fed into *tximport* function. Only gene-coding mRNA were selected (n=19,035), since the SCAN-B library preparation protocol enriched mRNA by poly-A tail selection.

Gene Set Enrichment Analysis (GSEA)

Input for the GSEA analysis consisted on gene-level statistics obtained from separate regression analysis in our discovery and validation dataset. P-values were combined using the Fisher method as implemented in the metaRNAseq R package.(5) Genes with low-gene count and conflicting differential expression (i.e. with opposite effect size direction) were excluded. In total 7,881 genes (3,679 with negative, and 4,202 with positive beta estimate) were included in the analysis. A total of 1,948 genes (801 down-regulated and 1,147 up-regulated) were mapped onto the MSigDB hallmark gene set collection.

We computed gene set-level statistics using six different GSEA methods that make use of all available gene-level statistics (P-value) obtained from the regression analysis, while considering the direction for association (i.e. negative or positive beta estimate): Wilcoxon rank-sum test, tail strength, mean, median, sum, reporter features, and Stouffer's method. For each of the methods, five gene set P-values were obtained under three directionality classes: non-directional, distinct-directional, and mixed-directional. In the former two, a P-value on each direction of association is computed. Under the 'distinct' directionality class, differential expression in opposite direction is allowed to counteract with each other, while under the 'mixed' directionality class, differential expression by sub-set of genes associated on either direction is tested on each gene set.

Gene set P-values were estimated based on the empirical background distribution generated through 10,000 permutations of gene labels. We controlled the FDR to be lower than 0.05. A consensus 'mean' score was computed for each possible directionality class in order to rank most significant gene sets, based on the adjusted P-values from the seven different GSA methods. To summarize statistically significant enrichment under each class, the median adjusted P-value was reported.

Supplementary Table 1. Descriptive table of risk factors for 2,474 LIBRO-1/KARMA participants.

Age at diagnosis in years, mean (SD)	57.8 (10.32)
Age at menarche in years, mean (SD)	13.08 (1.52)
Age at first child in years, mean (SD)	26.66 (5.32)
Menopause status, n (%)	
Premenopausal	273 (11.03)
Peri-menopausal	435 (17.58)
Postmenopausal	1766 (71.38)
Parity, n (%)	
None live-born children	396 (16.01)
One or more children	2,070 (83.67)
Unknown	8 (0.32)
Length in meters, mean (SD)	1.66 (0.06)
Weight in kilograms, mean (SD)	70.77 (12.21)
Body mass index in kg/m^2, mean (SD)	25.71 (4.23)
Hormone replacement therapy, n (%)	
No use	1,732 (70.01)
More than 5 years	261 (10.55)
Less than 5 years	220 (8.89)
Current use	261 (10.55)
Hyperplasia, n (%)	
Negative	2,344 (94.75)
Positive	130 (5.25)
Atypical hyperplasia, n (%)	
Negative	2,452 (99.11)
Positive	22 (0.89)
Lobular cancer in situ, n (%)	
Positive	0 (0.00)
Family history of breast cancer, n (%)	
Negative	1,917 (77.49)
Positive	472 (19.08)
Unknown	85 (3.44)

Supplementary Table 2. List of the 37 genes associated with breast cancer risk (TC score) in the discovery dataset, included in the low-risk TC-Gx.

ENSEMBL_GENE_ID	Name	GENEID	β	logCPM	P-value	FDR	β*	P-value*
ENSG00000198183	BPI fold containing family A member 1	BPIFA1	1.542	2.181	6.37E-21	1.58E-17	-0.269	2.86E-01
ENSG00000187733	amylase, alpha 1C	AMY1C	1.532	3.133	8.68E-10	4.29E-07	**0.841**	6.42E-03
ENSG00000171431	keratin 20	KRT20	1.326	4.239	1.26E-07	3.36E-05	-2.852	6.49E-17
ENSG00000167531	lactalbumin alpha	LALBA[Φ]	1.081	0.381	4.36E-15	5.82E-12	**2.298**	4.16E-15
ENSG00000181617	follicular dendritic cell secreted protein	FDCSP[Φ]	0.925	6.501	1.23E-05	1.86E-03	**1.149**	3.19E-08
ENSG00000096006	cysteine rich secretory protein 3	CRISP3	0.918	4.841	5.70E-09	2.15E-06	-0.632	9.05E-05
ENSG00000143556	S100 calcium binding protein A7	S100A7	0.911	6.308	9.24E-04	4.27E-02	-0.304	2.07E-01
ENSG00000171564	fibrinogen beta chain	FGB	0.779	3.969	2.38E-05	3.22E-03	-0.245	1.67E-01
ENSG00000215113	chromosome X open reading frame 49B	CXorf49B	0.769	0.477	1.24E-09	5.83E-07	**0.554**	5.83E-04
ENSG00000174876	amylase, alpha 1B	AMY1B	0.758	2.316	2.51E-06	4.82E-04	**0.478**	3.86E-02
ENSG00000124102	peptidase inhibitor 3	PI3	0.738	1.820	4.83E-04	2.81E-02	-0.773	1.45E-02
ENSG00000143546	S100 calcium binding protein A8	S100A8	0.677	6.377	2.11E-04	1.58E-02	-0.716	1.84E-05
ENSG00000170893	thyrotropin releasing hormone	TRH	0.668	3.691	2.53E-06	4.82E-04	**0.137**	6.34E-01
ENSG00000147255	immunoglobulin superfamily member 1	IGSF1	0.661	4.802	4.02E-12	3.03E-09	**0.322**	1.75E-01
ENSG00000166535	alpha-2-macroglobulin like 1	A2ML1[Φ]	0.654	3.806	5.09E-07	1.17E-04	**1.079**	6.80E-12
ENSG00000102854	mesothelin	MSLN	0.649	2.341	8.69E-07	1.88E-04	**0.510**	6.22E-05
ENSG00000171557	fibrinogen gamma chain	FGG	0.612	4.293	7.42E-04	3.69E-02	-0.345	2.37E-02
ENSG00000186832	keratin 16	KRT16	0.603	3.892	6.15E-05	6.73E-03	**0.515**	5.20E-04
ENSG00000164825	defensin beta 1	DEFB1	0.600	0.430	6.43E-04	3.41E-02	**0.135**	3.50E-01
ENSG00000135413	lacritin	LACRT	-0.586	3.271	7.93E-04	3.89E-02	0.440	1.19E-01
ENSG00000173114	leucine rich repeat neuronal 3	LRRN3	-0.686	3.651	2.18E-11	1.45E-08	0.032	7.28E-01
ENSG00000079689	secretagogin, EF-hand calcium binding protein	SCGN	-0.691	0.812	2.94E-09	1.21E-06	0.403	6.68E-02
ENSG00000180537	ring finger protein 182	RNF182	-0.708	2.154	4.19E-13	3.93E-10	0.314	2.59E-02
ENSG00000203837	pancreatic lipase related protein 3	PNLIPRP3[Φ]	-0.709	1.429	2.53E-06	4.82E-04	**-1.539**	1.54E-13
ENSG00000104321	transient receptor potential cation channel subfamily A member 1	TRPA1	-0.759	5.428	4.31E-13	3.93E-10	0.101	4.65E-01
ENSG00000205754	solute carrier organic anion transporter family member 1B7	SLCO1B7[Φ]	-0.809	1.462	1.02E-08	3.52E-06	**-1.818**	1.01E-09
ENSG00000171551	endothelin converting enzyme like 1	ECEL1	-0.814	2.970	2.06E-09	8.70E-07	0.141	6.83E-02
ENSG00000196228	sulfotransferase family 1C member 3	SULT1C3[Φ]	-0.817	0.704	6.65E-10	3.49E-07	**-2.349**	5.40E-08
ENSG00000163631	albumin	ALB	-0.842	4.690	2.03E-13	2.07E-10	**-0.323**	8.75E-02
ENSG00000266524	growth differentiation factor 10	GDF10	-0.859	1.949	4.48E-14	5.54E-11	**-0.002**	9.79E-01
ENSG00000110680	calcitonin related polypeptide alpha	CALCA	-0.885	0.812	8.03E-10	4.09E-07	1.518	5.35E-18
ENSG00000135226	UDP glucuronosyltransferase family 2 member B28	UGT2B28[ϕ]	-0.902	2.520	1.00E-05	1.62E-03	**-1.951**	1.19E-09
ENSG00000267978	MAGE family member A9B	MAGEA9B	-0.940	0.678	4.08E-15	5.82E-12	0.079	5.76E-01
ENSG00000114200	butyrylcholinesterase	BCHE	-1.057	3.436	4.33E-07	1.03E-04	0.656	5.57E-06
ENSG00000096088	progastricsin	PGC[Φ]	-1.212	1.586	1.16E-12	1.00E-09	**-0.909**	3.56E-09
ENSG00000198077	cytochrome P450 family 2 subfamily A member 7	CYP2A7	-1.690	3.693	6.57E-33	1.14E-28	**-0.141**	5.08E-01
ENSG00000237412	protease, serine 56	PRSS56	-2.046	2.419	7.00E-29	4.04E-25	0.765	1.72E-07

List of 37 genes found to be strongly correlated with 5-year TC score, as per 1-percent decrease on the TC scale. Genes were found associated with TC at FDR < 0.05 and beta coefficient (β) > $\pm\log_2(1.5)$ in the validation dataset, which are shown sorted by the β effect size. Validation (SCAN-B) effect estimates found in the same direction of association are marked in boldface type.

*, test statistics from an independent differential expression analysis in the validation dataset.

[Φ], genes replicated in the validation dataset at P-value < 1×10-5 and β > $\pm\log_2(1.5)$.

Supplementary Figure 1. Scatterplot showing correlation between the low-risk TC-Gx and the TC score in the discovery dataset. Regression line, beta coefficient (per one standard deviation (SD) in the low-risk TC-Gx), and P-value were obtained from an unconditional linear regression model.

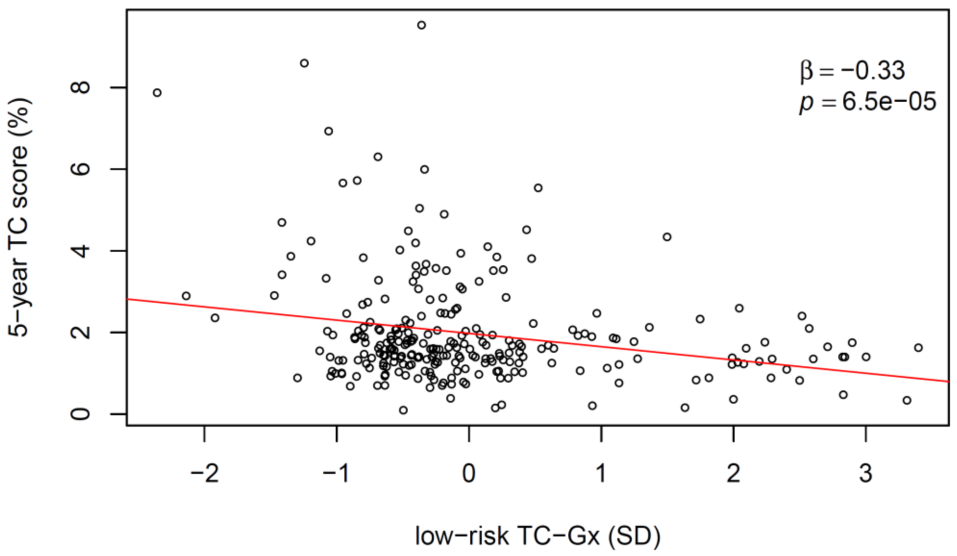

Supplementary references

1. Rantalainen M, Klevebring D, Lindberg J, Ivansson E, Rosin G, Kis L, et al. Sequencing-based breast cancer diagnostics as an alternative to routine biomarkers. Sci Rep-Uk. 2016;6.

2. Saal LH, Vallon-Christersson J, Hakkinen J, Hegardt C, Grabau D, Winter C, et al. The Sweden Cancerome Analysis Network - Breast (SCAN-B) Initiative: a large-scale multicenter infrastructure towards implementation of breast cancer genomic analyses in the clinical routine. Genome Med. 2015;7(1):20.

3. Patro R, Duggal G, Love MI, Irizarry RA, Kingsford C. Salmon provides fast and bias-aware quantification of transcript expression. Nat Methods. 2017;14(4):417-9.

4. Soneson C, Love MI, Robinson MD. Differential analyses for RNA-seq: transcript-level estimates improve gene-level inferences. F1000Res. 2015;4:1521.

5. Rau A, Marot G, Jaffrezic F. Differential meta-analysis of RNA-seq data from multiple studies. Bmc Bioinformatics. 2014;15.
